# Molecular Evolution of Human Norovirus GII.2 Clusters

**DOI:** 10.3389/fmicb.2021.655567

**Published:** 2021-03-22

**Authors:** Xingguang Li, Haizhou Liu, Brittany Rife Magalis, Sergei L. Kosakovsky Pond, Erik M. Volz

**Affiliations:** ^1^Department of Hospital Office, The First People’s Hospital of Fangchenggang, Fangchenggang, China; ^2^Centre for Emerging Infectious Diseases, The State Key Laboratory of Virology, Wuhan Institute of Virology, University of Chinese Academy of Sciences, Wuhan, China; ^3^Institute for Genomics and Evolutionary Medicine, Temple University, Philadelphia, PA, United States; ^4^MRC Centre for Global Infectious Disease Analysis, School of Public Health, Imperial College London, London, United Kingdom

**Keywords:** human norovirus, genetic diversity, positive selection, virus evolution, phylogenetic analyses

## Abstract

**Background:**

The human norovirus GII.2 outbreak during the 2016–2017 winter season was of unprecedented scale and geographic distribution.

**Methods:**

We analyzed 519 complete *VP1* gene sequences of the human norovirus GII.2 genotype sampled during the 2016–2017 winter season, as well as prior (dating back to 1976) from 7 countries. Phylodynamic analyses of these sequences were performed using maximum likelihood and Bayesian statistical frameworks in order to estimate viral evolutionary and population dynamics associated with the outbreak.

**Results:**

Our results revealed an increase in the genetic diversity of human norovirus GII.2 during the recent Asian outbreak and diversification was characterized by at least eight distinct clusters. Bayesian estimation of viral population dynamics revealed a highly fluctuating effective population size, increasing in frequency during the past 15 years.

**Conclusion:**

Despite an increasing viral diversity, we found no evidence of an elevated evolutionary rate or significant selection pressure in human norovirus GII.2, indicating viral evolutionary adaptation was not responsible for the volatility of or spread of the virus during this time.

## Introduction

Human norovirus (HuNoV) is a pathogenic agent that contributes substantially to the global burden of sporadic cases and outbreaks of acute gastroenteritis across all settings and age groups in humans (Ahmed et al., 2014). HuNoV is a non-enveloped, single-stranded, positive-sense RNA virus from the genus *Norovirus* in the family *Caliciviridae* (Glass et al., 2009). HuNoV is classified into at least 10 genogroups (GI–GX) and 48 genotypes, based on phylogenetic analyses of the capsid gene (Zheng et al., 2006; Vinje, 2015; Chhabra et al., 2019, 2020). Among these different genogroups, genotypes belonging to the GI, GII, and GIV are primarily responsible for the acute gastroenteritis cases in humans (Kroneman et al., 2013; Vinje, 2015). GI and GII strains can be further stratified into nine and twenty-six genotypes, respectively (Kroneman et al., 2013; Chhabra et al., 2019, 2020).

Genotype GII.2 strains recently emerged, causing large outbreaks of acute gastroenteritis in Japan and China during the 2016–2017 winter season, mainly in childcare centers (Ao et al., 2017, 2018; Hata et al., 2018; Nagasawa et al., 2018). Many other countries also reported GII.2-dominated HuNoV outbreaks during the 2016–2017 winter season (Bidalot et al., 2017; Niendorf et al., 2017; Tohma et al., 2017; Medici et al., 2018; Thanusuwannasak et al., 2018).

To gain a better understanding of genetic variation during the recent HuNoV epidemic, it is essential to analyze the *VP1* gene, which is crucial for viral attachment and entry, as well as the production of neutralizing antibodies (Prasad et al., 1999; Tan et al., 2004; Chakravarty et al., 2005). Therefore, in order to gain a more comprehensive understanding of the viral evolutionary factors responsible for the emergence and spread accompanying the recent HuNoV epidemic, we performed a detailed analysis of the complete *VP1* gene of the GII.2 strains with known sampling dates and geographic locations. Our findings suggest that close monitoring of the global spread of this emergent GII.2 strains is necessary for the prevention and mitigation of HuNoV acute gastroenteritis outbreaks.

## Materials and Methods

### Sequence Data Set

All available complete ORF2 region sequences (at least 1,626 bp in length) of GII.2 strains with known sampling date and geographic location were retrieved from GenBank^1^ on 28 April 2018. Sequences were aligned using MAFFT v7.409 (Katoh and Standley, 2013) and then adjusted manually in BioEdit v7.2.5 (Hall, 1999). The best-fit model of nucleotide substitution for this data set was identified according to the Bayesian information criterion in jModelTest v2.1.10 (Darriba et al., 2012). Given the phylogenetic scope of our analysis, we chose to exclude models that allowed a proportion of invariable sites (Jia et al., 2014). For this data set, the SYM + Γ_4_ model provided the best fit and was used in genetic distance calculations and tree reconstruction.

### Phylogenetic, Likelihood-Mapping, and Genetic Distance Analyses

To evaluate the phylogenetic signal for this data set, we performed a likelihood mapping analysis (Strimmer and von Haeseler, 1997) using TREE-PUZZLE v5.3.rc16 (Schmidt et al., 2002). We then inferred the phylogeny using maximum likelihood (ML) in PhyML v3.1 (Guindon and Gascuel, 2003). Bootstrap support values were calculated using 1,000 pseudoreplicates. Methods for identifying and defining phylogenetic clusters differ between studies. In the present study, we identify them on the basis of bootstrap support (cut-off of 99%) for groupings with more than two sequences. Genetic distances between and within clusters were calculated in MEGA v5.05 (Tamura et al., 2011) using the maximum composite likelihood model (Tamura et al., 2004) with 1,000 bootstrap replicates.

### Evolutionary Rate, Time Origin, and Past Population Dynamic Inferences

To evaluate the temporal structure of this data set, we plotted root-to-tip genetic distances against date of sampling within the ML tree using TempEst v1.5 (Rambaut et al., 2016). We also estimated the evolutionary rate and the time to the most recent common ancestor for this data set using least-squares dating in LSD v0.3beta (To et al., 2016), a Gamma-Poisson mixture model in treedater package (Volz and Frost, 2017), and ML dating in TreeTime package (Sagulenko et al., 2018), respectively. We then employed a Bayesian phylogenetic approach using Markov chain Monte Carlo (MCMC) sampling to estimate the rate of evolution and the time to the most recent common ancestor for all sequences in BEAST v1.8.2 (Drummond et al., 2012). Evolutionary rates were estimated using a strict molecular clock model. A non-parametric coalescent Bayesian skygrid tree prior model was employed for demographic inference. The overall evolutionary rate was given an uninformative continuous-time Markov chain (CTMC) reference prior. The MCMC analysis was run for 500 million steps, with samples drawn every 50,000 steps. The first 1,000 samples in each chain were removed as burn-in. All parameters had effective sample sizes greater than 200, which is indicative of sufficient sampling. We ran multiple chains to check for convergence to the target distribution using Tracer v1.7.1 (Rambaut et al., 2018). Trees were summarized as maximum clade credibility (MCC) trees using TreeAnnotator v1.8.2 after discarding the first 10% as burn-in, and then visualized and annotated in FigTree v1.4.3^2^. We also employed the skygrowth package in R to estimate the effective population size for the *VP1* gene of the HuNoV GII.2 strains (Volz and Didelot, 2018).

### Analysis of Selection

The evidence of gene-wide episodic positive selection along the backbone of the phylogenetic tree was performed using BUSTED, a test for episodic diversifying selection (Murrell et al., 2015), implemented in HyPhy software package (Pond et al., 2005). Initial branch lengths within the fixed MCC tree topology were estimated using the GTR (general time reversible) nucleotide model. A likelihood ratio test (LRT) was used to compare optimized likelihoods under the unconstrained, alternative model wherein the ratio of non-synonymous to synonymous rates (*dN/dS*) is allowed to exceed 1 (positive selection) and under the constrained (*dN/dS* = 1), null model (neutrality). A related test, RELAX (Wertheim et al., 2015) was used to investigate relaxation or intensification of selective regimes within each of the eight highly supported clades as compared to the persisting viral lineages. External branches were placed in the nuisance category to avoid *dN/dS* estimate inflation owing to transient polymorphisms. The selection intensity parameter (*k*) was estimated for each clade and the LRT used to compare the null model in which *k* is constrained to 1 (i.e., the same *dN/dS* distribution on test and reference branches) to an alternative model in which *k* is a free parameter for each clade. *p* ≤ 0.05 were considered significant evidence in favor of the alternative selection model for both tests.

## Results

### Sequence Data Set Information

This data set included 519 complete ORF2 region sequences of GII.2 strains from Australia (*n* = 3); the Mainland of China (*n* = 302); Hong Kong (*n* = 22); Japan (*n* = 166); Malaysia (*n* = 1); Netherlands (*n* = 10); Taiwan (*n* = 9); United Kingdom (*n* = 1); and United States (*n* = 5), with sampling dates between 1976 and 2017 (Table 1 and Supplementary Table S1).

**TABLE 1:** Geographic source and sampling year of human norovirus GII.2 sequences used in the present study.

	*n*	Sampling year				
	
Geographic source		1970–1979	1980–1989	1990–1999	2000–2009	2010–2017
Australia	3					3
China	302					302
Hong Kong	22					22
Japan	166			1	52	113
Malaysia	1	1				
Netherlands	10			1	9	
Taiwan	9					9
United Kingdom	1		1			
United States	5	1		1		3
**Total**	**519**	**2**	**1**	**3**	**61**	**452**

### Phylogenetic Analysis

**FIGURE 1:**
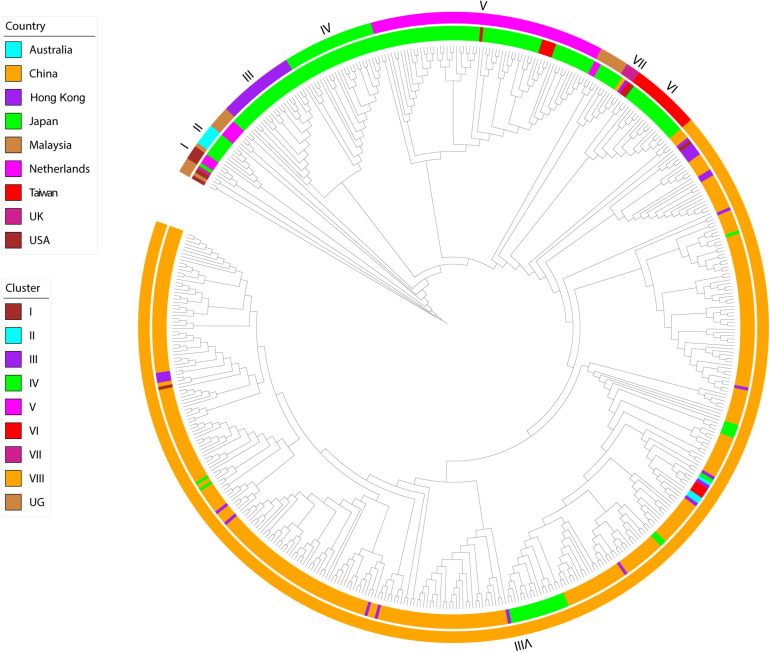
Maximum-likelihood phylogeny of HuNoV GII.2 strains. The two circles of color show geographic location (inner) and phylogenetic cluster (outer).

**FIGURE 2:**
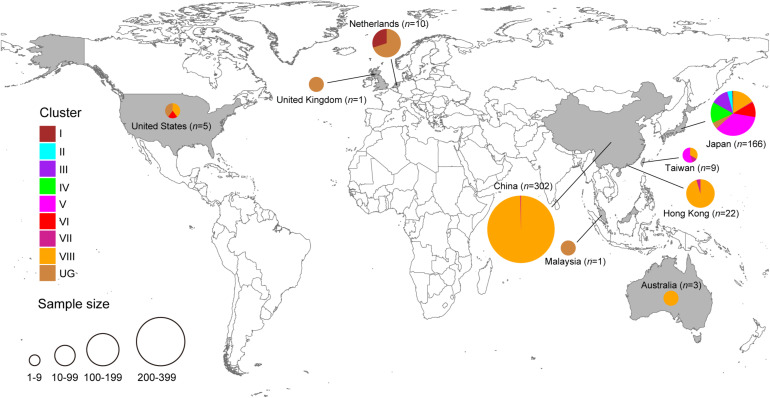
Geographic distribution of HuNoV GII.2 clusters identified in the present study. Each GII.2 cluster identified in this study is color-coded, as shown on the left. The figure was created using Adobe Illustrator CS5 version 15.0.0 software, based on the maps obtained from Craft MAP website (craftmap.box-i.net/).

The phylogeny of this data set, inferred using maximum likelihood, indicated the presence of eight transmission clusters (Figures 1, 2, Supplementary Figure S1, Table 2, and Supplementary Table S2). Sequences from Cluster I (*n* = 4) were found in Netherlands and Japan between 1999 and 2002. Cluster II sequences (*n* = 6) were found in Japan between 2000 and 2006. Sequences from Cluster III (*n* = 21) were collected from Japan between 2004 and 2006. Cluster IV sequences (*n* = 25) were found in Japan between 2007 and 2015, whereas Cluster V sequences (*n* = 64) were collected from Japan and Taiwan between 2008 and 2015. Cluster VI included eighteen sequences collected from Japan and one sequence collected from United States between 2010 and 2012. The four sequences from Cluster VII were collected from Japan, Taiwan, Hong Kong, and the Mainland of China between 2015 and 2017. Cluster VIII sequences (*n* = 357) were found in Australia, the Mainland of China, Hong Kong, Japan, Taiwan, and United States between 2016 and 2017.

**TABLE 2:** Classification and geographic source of human norovirus GII.2 sequences used in the present.

Cluster	*n*	Geographic source								
	
		Australia	China	Hong Kong	Japan	Malaysia	Netherlands	Taiwan	United Kingdom	United States
I	4				1		3			
II	6				6					
III	21				21					
IV	25				25					
V	64				59			5		
VI	19				18					1
VII	4		1	1	1			1		
VIII	357	3	301	21	27			3		2
Ungrouped	19				8	1	7		1	2
**Total**	**519**	**3**	**302**	**22**	**166**	**1**	**10**	**9**	**1**	**5**

The remaining 19 sequences (designated as Ungrouped) were scattered throughout the main GII.2 genotype and had been sampled in Japan (*n* = 8), Malaysia (*n* = 1), Netherlands (*n* = 7), United Kingdom (*n* = 1), and United States (*n* = 2) between 1976 and 2014.

Of the 302 Chinese strains in our analysis, all were found within either cluster VII (*n* = 1) or cluster VIII (*n* = 301) and all were sampled within either 2016 (*n* = 133) or 2017 (*n* = 169). However, the 166 Japanese strains were interspersed throughout the phylogeny and were sampled within a wide range (1997, 2000, 2002, and 2004–2016.

### Likelihood-Mapping and Evolutionary Divergence Analysis

Our likelihood-mapping analysis revealed significant differences in phylogenetic signal and the amount of evolutionary information between each cluster in our data set (Supplementary Figure S2). The most notable result of the likelihood-mapping analysis revealed that 45.4% of the quartets from cluster III were distributed in the center of the triangle, indicating a relative strong star-like phylogenetic signal reflecting a new cluster, which might be due to exponential epidemic spread. Intriguingly, cluster III was characterized by the lowest genetic divergence among the eight distinct HuNoV GII.2 clusters (Supplementary Figure S3), which further confirmed that cluster III was most likely a new cluster, in accordance with likelihood-mapping analysis. Likewise, 38.5% of the quartets from cluster VI were distributed in the center of the triangle and showed lower genetic divergence. Conversely, 12.3% of the quartets from cluster VIII were distributed in the center of the triangle and showed higher genetic divergence than cluster VI. Notably, 43.5% of the quartets from cluster IV were distributed in the center of the triangle, but with higher genetic divergence than cluster VI. In contrast, 0, 0, 3.9, and 0% of the quartets from clusters I, II, V, and VII were distributed in the center of the triangle, but with higher genetic divergence, indicating the presence of a strong phylogenetic signal in the four clusters. Also of note, 3.4% of the quartets from the overall epidemics were distributed in the center of the triangle, but with lower genetic divergence (4.1%). We also observed that the smallest genetic distance separated clusters III and IV (3.9%), whereas the largest was between clusters I and VIII (10.1%). Notably, all the genetic distances between each of the eight clusters are higher than within each of the eight clusters, except for the genetic distance between clusters III and IV. We can also observe mixed genetic distances between and within each of the eight clusters (Supplementary Figure S3).

### Evolutionary Rate and Time Origin Analyses

**FIGURE 3:**
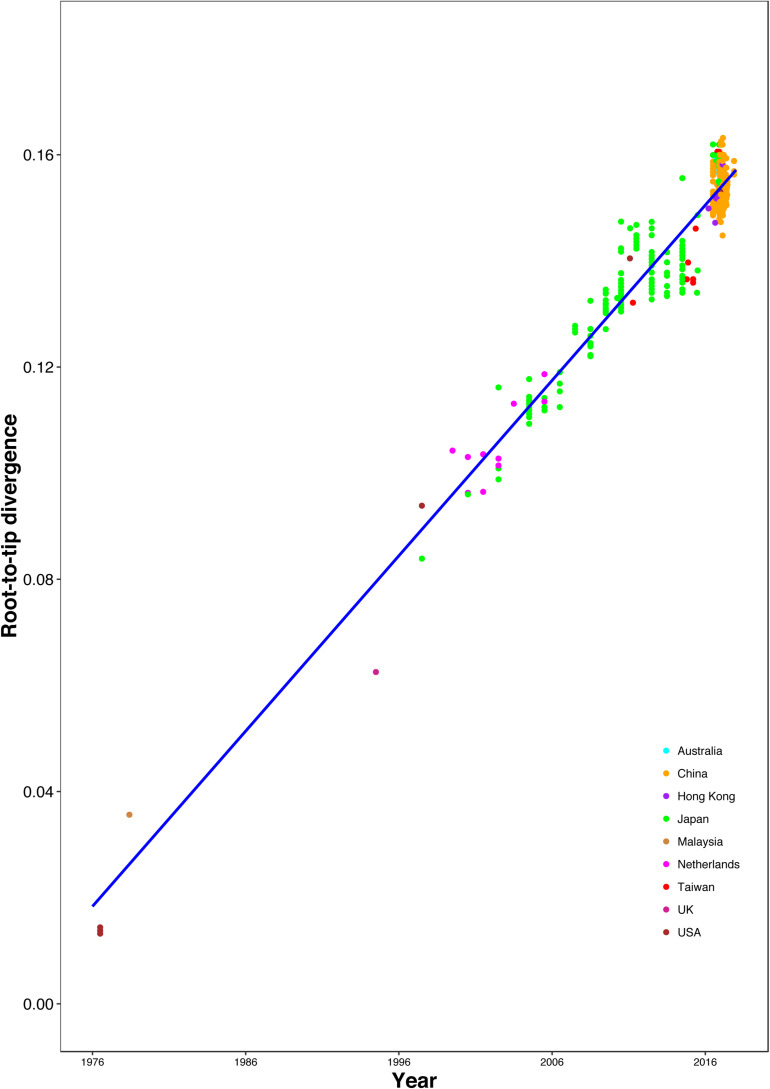
Regression of the root-to-tip genetic distance against year of sampling for the HuNoV GII.2 sequences. Colors indicate different geographic locations.

The correlation between root-to-tip distances and sampling time indicated a strong temporal signal (*R*^2^ = 0.9359), without any clear outlier sequences (Figure 3). This result suggests a relatively clocklike pattern of molecular evolution, with an estimated evolutionary rate of 3.29 × 10^–3^ substitutions per site per year and the most recent common ancestor occurring in 1970.37. We also analyzed the correlation between genetic distances and sampling time intervals, which indicated a strong linear regression signal for sampling time intervals > 12 years (Supplementary Figure S4). The estimated evolutionary rate and the time to the most recent common ancestor using a Gamma-Poisson mixture model were 2.76 × 10^–3^ substitutions per site per year and 1968.60, respectively. The estimated evolutionary rate and the time to the most recent common ancestor using maximum likelihood dating were 2.48 × 10^–3^ substitutions per site per year (95% credibility interval: 2.18 × 10^–3^–2.78 × 10^–3^) and 1969.0 (95% credibility interval: 1966.35–1971.65), respectively. The estimated evolutionary rate and the time to the most recent common ancestor using least-squares dating were 2.63 × 10^–3^ substitutions per site per year (95% credibility interval: 2.30 × 10^–3^–2.82 × 10^–3^) and 1968.61 (95% credibility interval: 1965.27–1970.84), respectively. In our Bayesian phylogenetic analysis, we estimated a evolutionary rate of 3.00 × 10^–3^ substitutions per site per year (95% credibility interval: 2.67 × 10^–3^–3.35 × 10^–3^). And the time to the most recent common ancestor of 1968.89 (95% credibility interval: 1965.49–1972.06). The age of each GII.2 cluster was also estimated in the analysis (Supplementary Table S3 and Supplementary Figure S5). The divergences between the sequences from clusters VI and VIII were estimated to have occurred in 2007.96 (95% credibility interval: 2007.10–2008.75) and 2012.39 (95% credibility interval: 2011.42–2013.44), respectively. And the time to the most recent common ancestor of 2004.61 (95% credibility interval: 2003.22–2005.83) for clusters VI and VIII.

### Analysis of Past Population Dynamics

**FIGURE 4:**
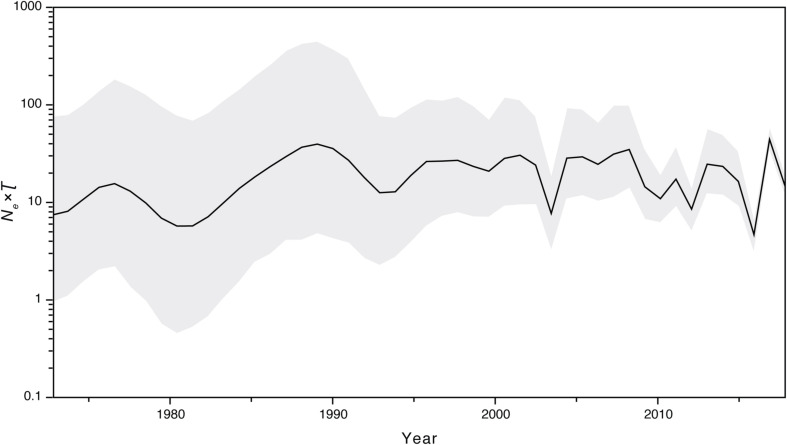
Bayesian skygrid demographic reconstruction of HuNoV GII.2. The vertical axis shows the effective number of infections (*N*_*e*_) multiplied by mean viral generation time (τ). The solid line and shaded region represent the median and 95% credibility interval, respectively, of the inferred *N*_*e*_τ through time.

**FIGURE 5:**
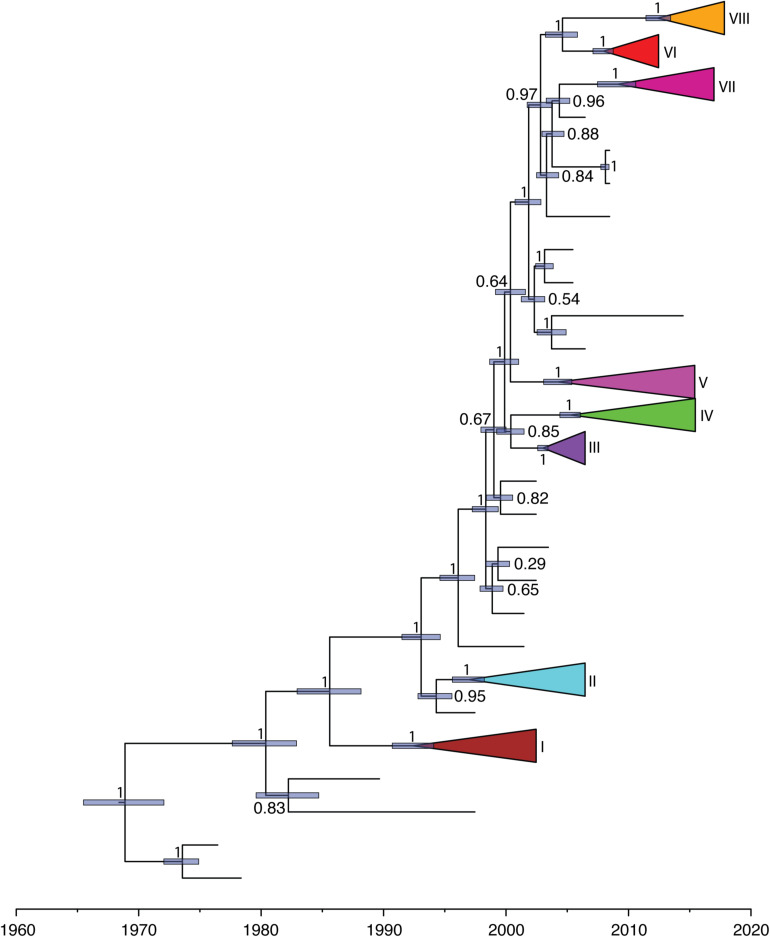
Maximum-clade-credibility tree estimated from complete *VP1* gene sequences of HuNoV GII.2. Light blue horizontal bars represent 95% credibility intervals for estimates of node times. Posterior probabilities are shown at the node. Each cluster is shown with different colors.

We further investigated the past population dynamics of this data set using a Bayesian skygrid plot. The effective population size of the *VP1* gene in the HuNoV GII.2 strains experienced very high volatility of population size, especially for the past 15 years (Figure 4). The effective population size of the *VP1* gene in the HuNoV GII.2 strains was also estimated using MCMC method in skygrowth package (Supplementary Figure S6), the estimates of the effective population size were consistent with the Bayesian skygrid model. The estimates of the phylogenetic relationships among the HuNoV GII.2 sequences using Bayesian coalescent framework were consistent with maximum likelihood tree reconstruction (Figure 5).

### Analysis of Selection

Given the observed variations in evolutionary dynamics for this data set, we sought to investigate the selective pressures driving the persistence of HuNoV GII.2. No evidence of episodic positive selection was detected among the persisting viral lineages (Supplementary Table S4, *p* = 0.18); however, significant differences in selective strength among outbreaks when compared to these persisting lineages were found (*p* = 0.04). Clusters II, III, V, and VII exhibited intensified selection (*k* > 2), whereas sister clusters VI and VIII were considered to have relaxed in selective strength (*k* < 0.5). Clusters I and IV were characterized by relatively similar selective regimes in comparison to the backbone (*k* = 0.72 and 0.6, respectively). The results suggested that the evolutionary dynamics of the *VP1* gene of HuNoV GII.2 strains in this data set were influenced largely by natural evolutionary forces, rather than adaptation to selective pressure from the host immune system, which was consistent with the previous study (Tohma et al., 2017).

## Discussion

Our evolutionary analyses, based on all of the available complete *VP1* sequences of HuNoV GII.2 genotype that included country of origin and year of sampling, revealed the presence of at least eight independent clusters. Cluster VII was restricted to countries within Asia, whereas cluster VIII had dispersed to Asia and North America. The recent sequences from cluster VIII outbreak formed a highly distinct sister group to cluster VII. As such, the putative HuNoV GII.2 reservoirs for cluster VIII outbreak sequences might be distinct from that of cluster VII.

Our estimates of the substitution rate for this data set were reassuringly consistent across different methods and models. The mean estimated rates of HuNoV GII.2 molecular evolution using TempEst v1.5 and BEAST v1.8.2 were 3.00 × 10^–3^ and 3.29 × 10^–3^ substitutions per site per year, respectively. These were also similar to the mean estimates previously reported for HuNoV GII.2 using BEAST v2.4.5, which was 3.26 × 10^–3^ substitutions per site per year. However, the mean estimated rates of HuNoV GII.2 molecular evolution using LSD v0.3beta, TreeTime package, and treedater package were 2.63 × 10^–3^, 2.48 × 10^–3^, and 2.76 × 10^–3^ substitutions per site per year, respectively, which were lower than the results using TempEst v1.5 and BEAST v1.8.2. In summary, despite the extensive and prolonged human-to-human transmission in the recent HuNoV GII.2 outbreak, HuNoV GII.2 was not mutating at an unusually elevated rate. Thus, we found little evolutionary signal that the virus has enhanced its virulence and/or transmissibility in humans during the recent HuNoV GII.2 outbreak. Coalescent-based demographic inference revealed a rapidly increasing population size for HuNoV GII.2 from late 2015 to early 2017 (Figure 4), consistent with the outbreak during this period of time.

Our analyses of selection did not reveal episodic positive selection representative of continual adaptation, nor was the more recent human norovirus GII.2 outbreak characterized by intensified selective pressure. Thus, the results from the present study seem to be consistent with earlier reports (Kobayashi et al., 2016; Parra et al., 2017; Nagasawa et al., 2018) and suggest that the recent evolution of HuNoV GII.2 has been dominated by genetic drift. Furthermore, the results also suggested a relationship between selection strength variation and geographical spread, as Clusters VI and VIII were the only clusters to contain sequences outside of Asia after 2010.

Our study has several limitations. First, our analysis was based on all of the available complete *VP1* sequences of HuNoV GII.2 genotype that included country of origin and year of sampling, and we did not randomly collected sequences, we may therefore have underestimated the total number of infections. Second, the number of sample size is limited, which may provide an incomplete picture of phylogenetic relationship of the virus, therefore, which may have underestimated the total number of phylogenetic clusters. Additional sequences are critically needed to investigate the phylogenetic cluster changes over time, estimate its date of emergence, monitor local transmission, and infer past population dynamics of the virus. Additional sequences will also help to reveal the presence of additional phylogenetic clusters. Third, there are probably biases in geographic and temporal distributions of the *VP1* sequences presented in the present study, given that our study relies on data from different countries or stages that is varied significantly, which may introduce bias our estimates. Despite the aforementioned limitations, we believe our findings are reliable and robust.

Taken together, we analyzed 519 complete *VP1* gene sequences of the human norovirus GII.2 genotype. Most of the samples were collected during the outbreak during the 2016–2017 winter season from the Mainland of China. We explored several aspects about geographic distribution, demographic analysis and selective pressure of the GII.2 norovirus strains. This information is important for surveillance of viral strains with pandemic potential. Based on the phylogenetic analysis of these sequences, we showed an increase in the genetic diversity of human norovirus GII.2 for at least eight distinct clusters, and the geographic and gene divergence varied among clusters. Our results sufficiently demonstrated an increase in the genetic diversity of human norovirus GII.2 during the recent Asian outbreak and a highly fluctuating effective population size. We also indicated that viral evolutionary adaptation was not responsible for the volatility of or spread of the virus during this time. Our results also emphasize the importance of ongoing genetic and demographic surveillance of the epidemic, especially, sampled during the epidemic and across all the geographical range of the virus. These efforts, combined with epidemiological investigation, clinical recognition, and population movement data, are required to trace the history of recent epidemic waves and to assess adaptability and selection in HuNoV GII.2 strains. This is increasingly important for surveillance of viral strains with pandemic potential, and will further guide research on vaccines and therapeutic targets.

## Data Availability Statement

The original contributions presented in the study are included in the article/Supplementary Material, further inquiries can be directed to the corresponding author/s.

## Author Contributions

XL conceived and designed the study, performed the experiments, and drafted the manuscript. XL, BR, and EV analyzed the data. XL, HL, BR, EV, and SK interpreted data and provided critical comments. All authors reviewed and approved the final manuscript.

## Conflict of Interest

The authors declare that the research was conducted in the absence of any commercial or financial relationships that could be construed as a potential conflict of interest.

## Footnotes

1 http://www.ncbi.nlm.nih.gov/

2 http://tree.bio.ed.ac.uk/software/figtree

## Supplementary Material

Supplementary Figure 1 Phylogenetic relationships of complete *VP1* gene sequences from HuNoV GII.2. The tree was inferred using a maximum-likelihood analysis of 519 complete *VP1* gene sequences. Bootstrap values were shown at the nodes. Clusters were shown with different colors. The scale bar showed nucleotide substitutions per site.

Supplementary Figure 2 Likelihood-mapping analyses of complete *VP1* gene sequences from HuNoV GII.2. Likelihood-mapping analyses were performed for this data set, and also each of eight clusters defined by the maximum-likelihood phylogenetic analyses of complete *VP1* gene sequences from HuNoV GII.2.

Supplementary Figure 3 Average pairwise genetic distances within and between HuNoV GII.2 clusters using ggplot2 package. Green and orange colors indicated within and between human norovirus GII.2 clusters.

Supplementary Figure 4 Sampling time interval with respective genetic distance between pairs of HuNoV GII.2 clusters. Green and orange colors indicated within and between HuNoV GII.2 clusters.

Supplementary Figure 5 Estimated time to the most recent common ancestor of each HuNoV GII.2 cluster. Clusters were shown with different colors.

Supplementary Figure 6 Estimated effective population size through time using Bayesian Gibbs-within-Metropolis MCMC and fast maximum *a posteriori* algorithm. Estimated effective population size through time was based on the tree in Supplementary Figure 1.
