# Supplementary figures and images for: Molecular Evolution of Human Norovirus GII.2 Clusters
Source: Front Microbiol. 2021 Mar 22;12:655567. doi: 10.3389/fmicb.2021.655567 (PMC8019798; doi:10.3389/fmicb.2021.655567)

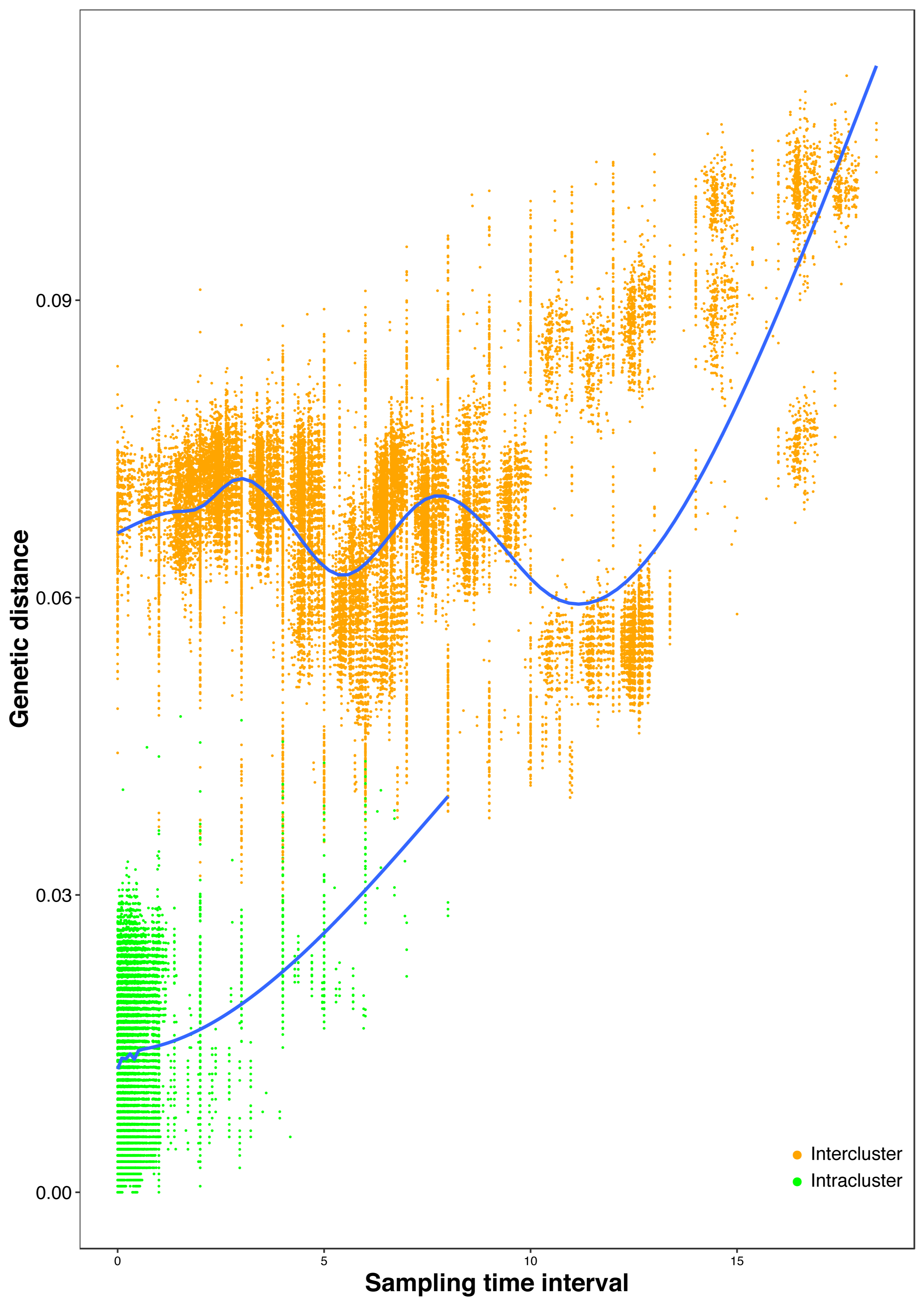

Supplement: Supplementary Figure 1 — Phylogenetic relationships of complete VP1 gene sequences from HuNoV GII.2. The tree was inferred using a maximum-likelihood analysis of 519 complete VP1 gene sequences. Bootstrap values were shown at the nodes. Clusters were shown with different colors. The scale bar showed nucleotide substitutions per site. [file Data_Sheet_1.zip › Fig.S4.tif]

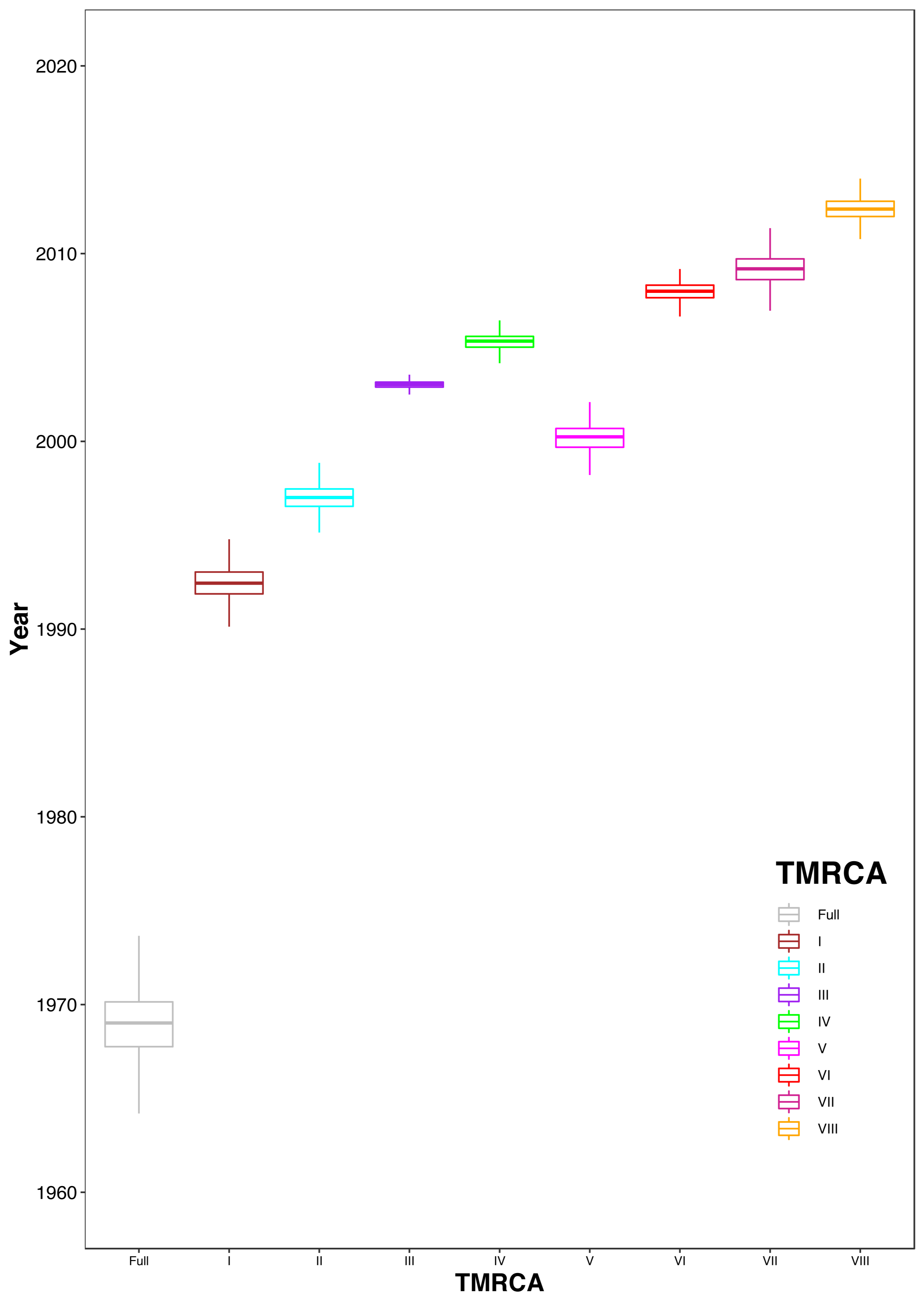

Supplement: Supplementary Figure 1 — Phylogenetic relationships of complete VP1 gene sequences from HuNoV GII.2. The tree was inferred using a maximum-likelihood analysis of 519 complete VP1 gene sequences. Bootstrap values were shown at the nodes. Clusters were shown with different colors. The scale bar showed nucleotide substitutions per site. [file Data_Sheet_1.zip › Fig.S5.tif]

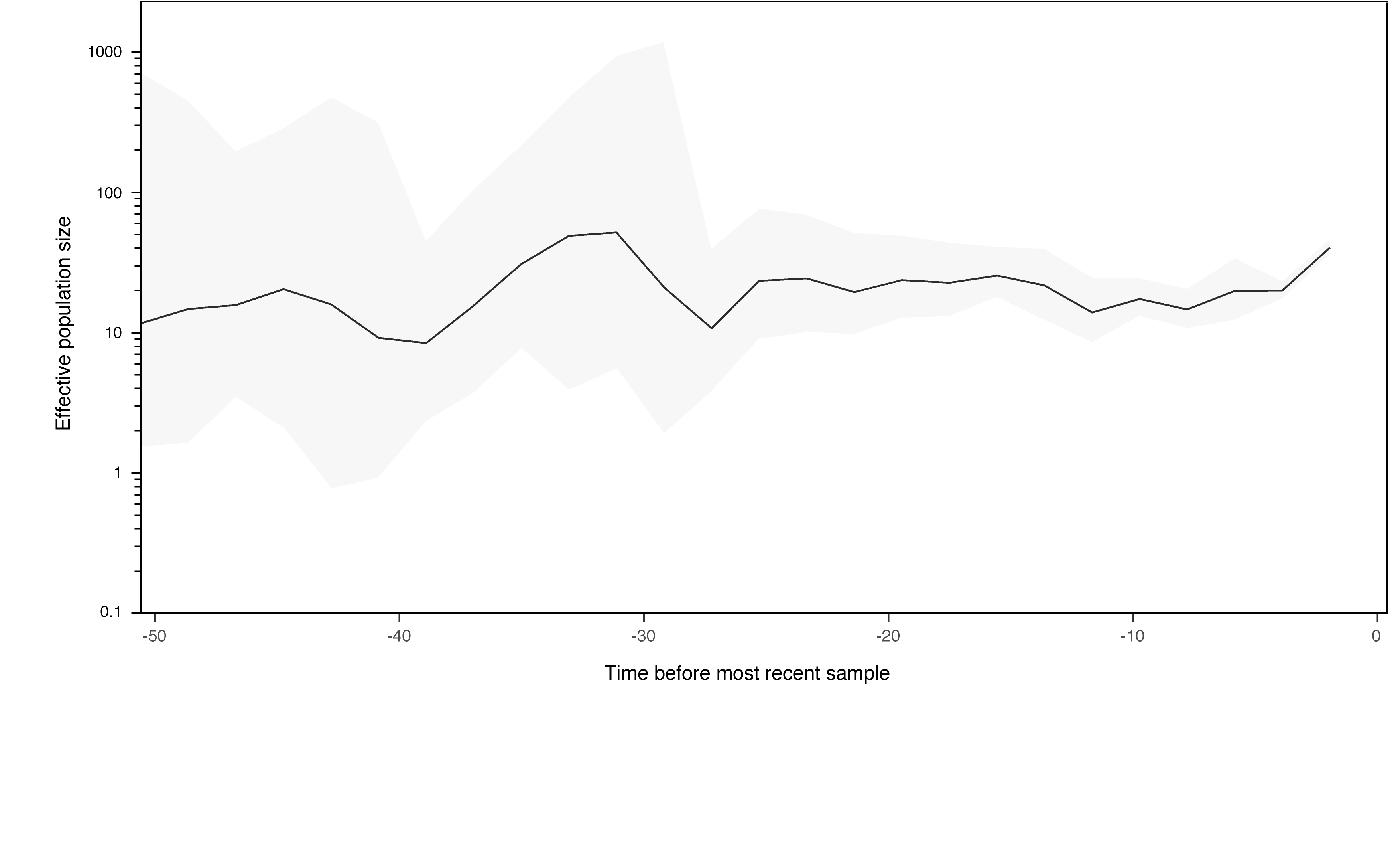

Supplement: Supplementary Figure 1 — Phylogenetic relationships of complete VP1 gene sequences from HuNoV GII.2. The tree was inferred using a maximum-likelihood analysis of 519 complete VP1 gene sequences. Bootstrap values were shown at the nodes. Clusters were shown with different colors. The scale bar showed nucleotide substitutions per site. [file Data_Sheet_1.zip › Fig.S6.tif]

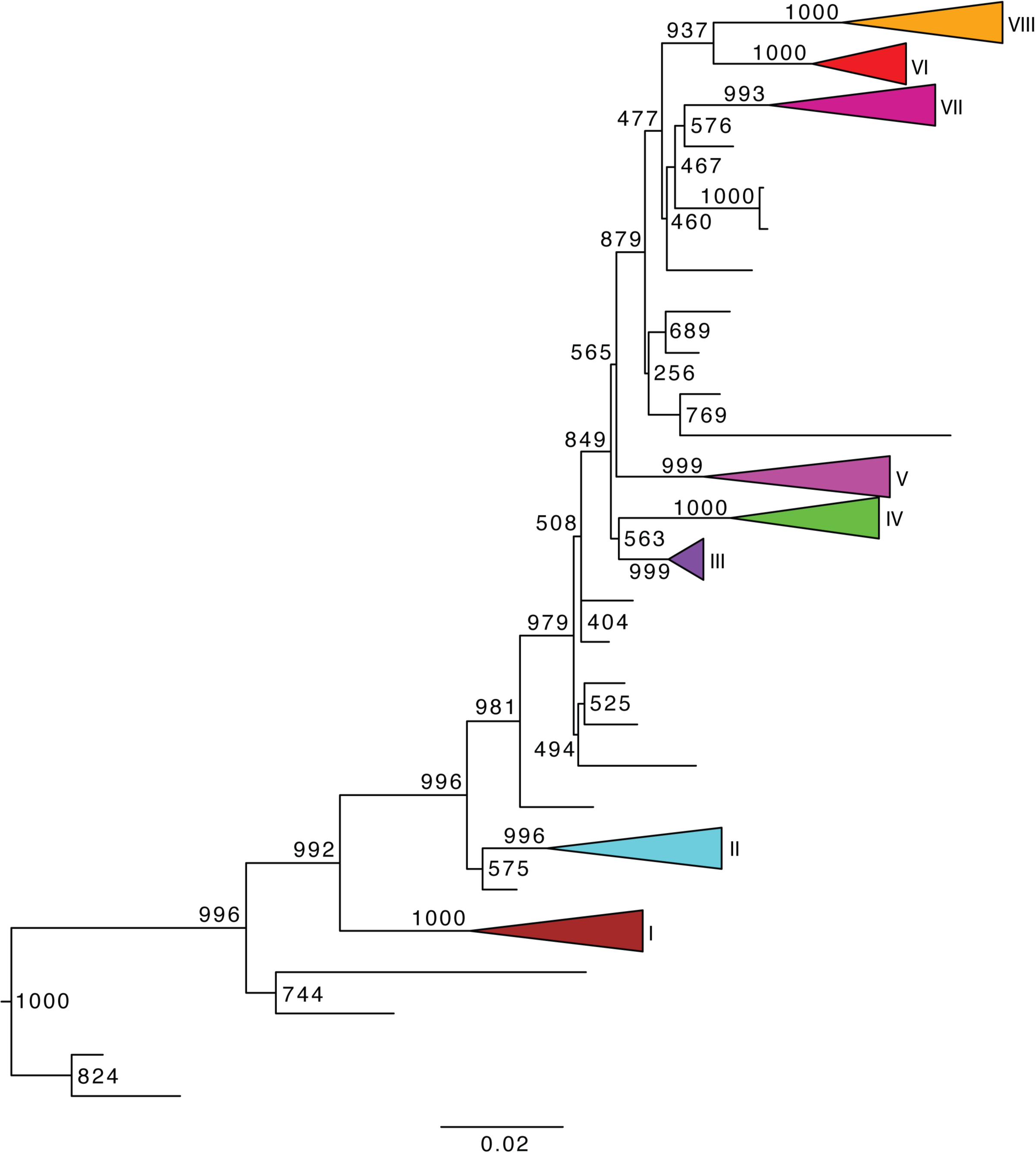

Supplement: Supplementary Figure 1 — Phylogenetic relationships of complete VP1 gene sequences from HuNoV GII.2. The tree was inferred using a maximum-likelihood analysis of 519 complete VP1 gene sequences. Bootstrap values were shown at the nodes. Clusters were shown with different colors. The scale bar showed nucleotide substitutions per site. [file Data_Sheet_1.zip › Fig.S1.tif]

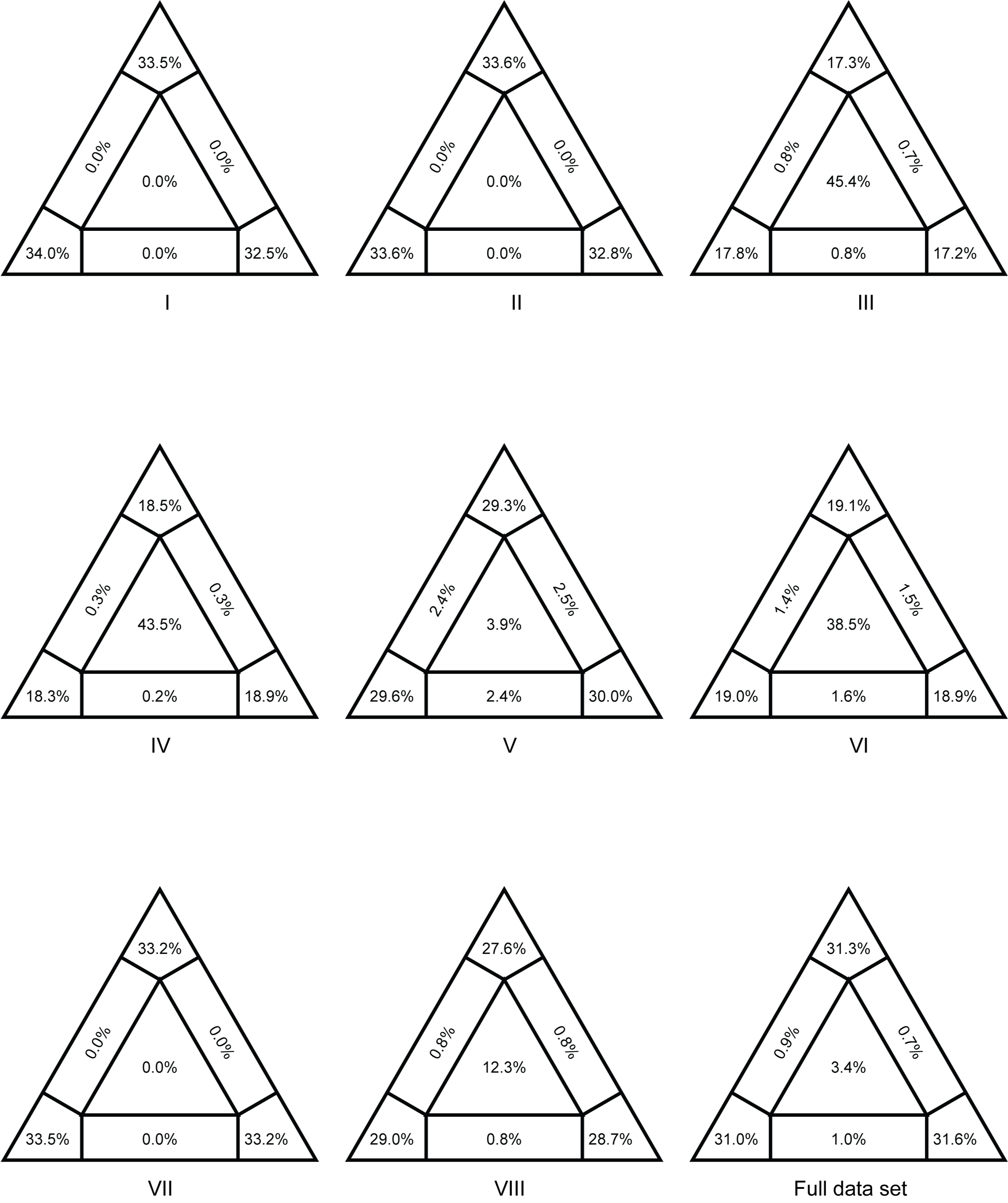

Supplement: Supplementary Figure 1 — Phylogenetic relationships of complete VP1 gene sequences from HuNoV GII.2. The tree was inferred using a maximum-likelihood analysis of 519 complete VP1 gene sequences. Bootstrap values were shown at the nodes. Clusters were shown with different colors. The scale bar showed nucleotide substitutions per site. [file Data_Sheet_1.zip › Fig.S2.tif]

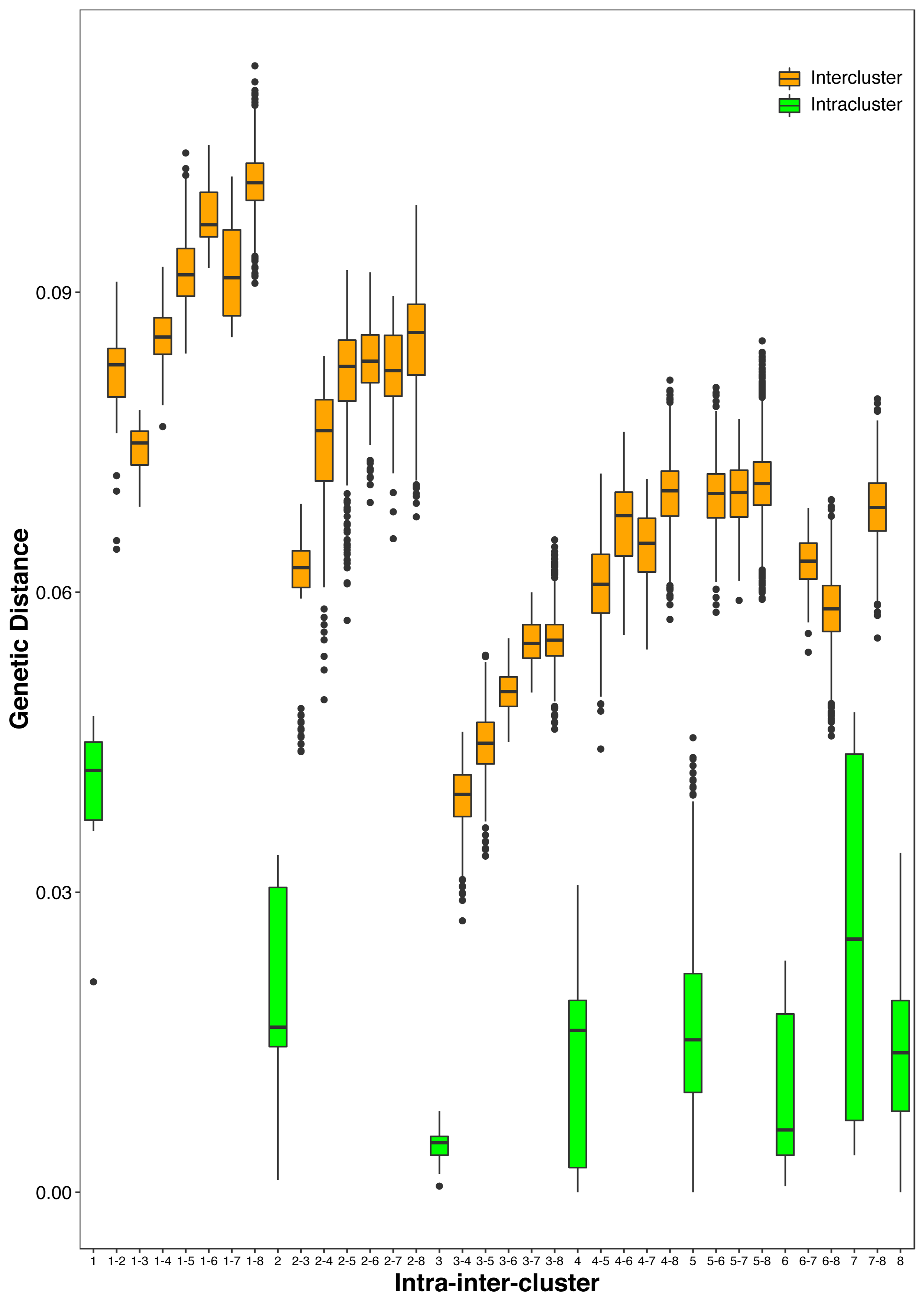

Supplement: Supplementary Figure 1 — Phylogenetic relationships of complete VP1 gene sequences from HuNoV GII.2. The tree was inferred using a maximum-likelihood analysis of 519 complete VP1 gene sequences. Bootstrap values were shown at the nodes. Clusters were shown with different colors. The scale bar showed nucleotide substitutions per site. [file Data_Sheet_1.zip › Fig.S3.tif]
