# Supplementary material for: Molecular Evolution of Human Norovirus GII.2 Clusters
Source: Front Microbiol. 2021 Mar 22;12:655567. doi: 10.3389/fmicb.2021.655567 (PMC8019798; doi:10.3389/fmicb.2021.655567)
Supplement: Supplementary file 2 [file Table_1.pdf]

Supplementary Table S1. List of *VP1* sequences of human norovirus GII.2 genotype used in the present study.

Accession number	Strain/Isolate name	Collection Country	Collection date
AY134748	Snow Mountain strain	United States	1976
JX846925	Hu/GII.2/KL109/1978/MYS	Malaysia	5/30/78
X81879	Melksham	United Kingdom	1989/9
AB279553	Hu/OC97049/1997/JP	Japan	1997
AY054300	NLV/Chesterfield/434/1997/US	United States	1997
AB281081	Hu/GII.2/Coevorden191S/1999/NL	Netherlands	1999
AB281085	Hu/GII.2/Zwolle25E/2001/NL	Netherlands	2001
AB281084	Hu/GII.2/Leeuwarden15/2001/NL	Netherlands	2001
EF547398	Hu/Maizuru/000602/2000/JP/2840	Japan	2000
AB281083	Hu/GII.2/Delft48M/2000/NL	Netherlands	2000
AB281082	Hu/GII.2/DenHaag37/2000/NL	Netherlands	2000
AB281087	Hu/GII.2/Rotterdam39E/2002/NL	Netherlands	2002
AB195225	Hu/NV/GII/Ina/02/JP	Japan	2002
AB279570	Hu/OCS020289/2002/JP	Japan	2002
AB281086	Hu/GII.2/Heerlen7E/2002/NL	Netherlands	2002
AB279555	Hu/OC02012/2002/JP	Japan	2002
AB281088	Hu/GII.2/Leeuwarden71/2003/NL	Netherlands	2003
LC209438	Hu/GII/JP/2004/GII.P2_GII.2/Tochigi-86	Japan	2004
LC209436	Hu/GII/JP/2004/GII.P2_GII.2/Tochigi-85	Japan	2004
LC209464	Hu/GII/JP/2004/GII.P2_GII.2/Hokkaido-13	Japan	2004
AB279564	Hu/OC04071/2004/JP	Japan	2004
AB279563	Hu/OC04067/2004/JP	Japan	2004
AB279572	Hu/OCS040035/2004/JP	Japan	2004
AB279573	Hu/OCS040100/2004/JP	Japan	2004
AB279562	Hu/OC04059/2004/JP	Japan	2004
AB279571	Hu/OCS030697/2004/JP	Japan	2004
AB279566	Hu/OC04075/2004/JP	Japan	2004
AB279565	Hu/OC04073/2004/JP	Japan	2004
AB279560	Hu/OC04056-1/2004/JP	Japan	2004
AB279561	Hu/OC04056-2/2004/JP	Japan	2004
AB279567	Hu/OC04076/2004/JP	Japan	2004
AB279557	Hu/OC04038/2004/JP	Japan	2004
AB279558	Hu/OC04042/2004/JP	Japan	2004
LC209437	Hu/GII/JP/2004/GII.P2_GII.2/Tochigi-87	Japan	2004
DQ456824	Hu/MK04/2004/JP	Japan	2004
AB279568	Hu/OC04169/2004/JP	Japan	2004
LC209435	Hu/GII/JP/2004/GII.P12_GII.2/Tochigi-92	Japan	2004
AB662852	Hu/GII.2/OC05143/2005/JP	Japan	2005
AB662853	Hu/GII.2/OC05145/2005/JP	Japan	2005
AB281090	Hu/GII.2/Vaals87/2005/NL	Netherlands	2005
AB281089	Hu/GII.2/Goes28/2005/NL	Netherlands	2005
AB662850	Hu/GII.2/OC05041/2005/JP	Japan	2005
AB662854	Hu/GII.2/OC06005/2006/JP	Japan	2006
LC209462	Hu/GII/JP/2006/GII.P2_GII.2/Hokkaido-14	Japan	2006
AB662863	Hu/GII.2/OH06023/2006/JP	Japan	2006

AB662856	Hu/GII.2/OC06014/2006/JP	Japan	2006
AB662864	Hu/GII.2/OH07001/2007/JP	Japan	2007
AB662858	Hu/GII.2/OC07107/2007/JP	Japan	2007
AB662865	Hu/GII.2/OH07013/2007/JP	Japan	2007
AB662868	Hu/GII.2/OH08020/2008/JP	Japan	2008
AB662861	Hu/GII.2/OC08154/2008/JP	Japan	2008
LC209463	Hu/GII/JP/2008/GII.P2_GII.2/Hokkaido-15	Japan	2008
AB662867	Hu/GII.2/OH08019/2008/JP	Japan	2008
AB662859	Hu/GII.2/OC08079/2008/JP	Japan	2008
AB662869	Hu/GII.2/OH08029-2/2008/JP	Japan	2008
AB662860	Hu/GII.2/OC08124/2008/JP	Japan	2008
AB662866	Hu/GII.2/OH08009/2008/JP	Japan	2008
AB662872	Hu/GII.2/OC09103/2009/JP	Japan	2009
AB662886	Hu/GII.2/OH09035/2009/JP	Japan	2009
AB662873	Hu/GII.2/OC09104/2009/JP	Japan	2009
AB662883	Hu/GII.2/OH09030/2009/JP	Japan	2009
AB662871	Hu/GII.2/OC09094/2009/JP	Japan	2009
AB662885	Hu/GII.2/OH09034/2009/JP	Japan	2009
AB662870	Hu/GII.2/OC09072/2009/JP	Japan	2009
LC209461	Hu/GII/JP/2009/GII.P16_GII.2/Kanagawa-49	Japan	2009
AB662881	Hu/GII.2/OH09028/2009/JP	Japan	2009
AB662884	Hu/GII.2/OH09032/2009/JP	Japan	2009
AB662892	Hu/GII.2/OH10011-2/2010/JP	Japan	2010
LC209474	Hu/GII/JP/2010/GII.P2_GII.2/Hiroshima-17	Japan	2010
LC209472	Hu/GII/JP/2010/GII.P2_GII.2/Hiroshima-18	Japan	2010
AB662898	Hu/GII.2/OH10024/2010/JP	Japan	2010
AB662901	Hu/GII.2/OH10029/2010/JP	Japan	2010
LC209473	Hu/GII/JP/2010/GII.P2_GII.2/Hiroshima-19	Japan	2010
LC209465	Hu/GII/JP/2010/GII.P2_GII.2/Hokkaido-16	Japan	2010
AB662888	Hu/GII.2/OH10005-2/2010/JP	Japan	2010
AB662891	Hu/GII.2/OH10008/2010/JP	Japan	2010
AB662879	Hu/GII.2/OC10026/2010/JP	Japan	2010
AB662899	Hu/GII.2/OH10025/2010/JP	Japan	2010
AB662893	Hu/GII.2/OH10012/2010/JP	Japan	2010
AB662894	Hu/GII.2/OH10013/2010/JP	Japan	2010
AB662889	Hu/GII.2/OH10006/2010/JP	Japan	2010
AB662876	Hu/GII.2/OC10012-2/2010/JP	Japan	2010
AB662896	Hu/GII.2/OH10020/2010/JP	Japan	2010
LC209454	Hu/GII/JP/2010/GII.P16_GII.2/Osaka-019	Japan	2010
AB662895	Hu/GII.2/OH10015-2/2010/JP	Japan	2010
AB662897	Hu/GII.2/OH10021/2010/JP	Japan	2010
AB662900	Hu/GII.2/OH10026/2010/JP	Japan	2010
AB662880	Hu/GII.2/OC10058/2010/JP	Japan	2010
LC209480	Hu/GII/JP/2010/GII.P16_GII.2/Ehime-44	Japan	2010
LC209481	Hu/GII/JP/2010/GII.P16_GII.2/Ehime-43	Japan	2010
LC209459	Hu/GII/JP/2010/GII.P16_GII.2/Kanagawa-51	Japan	2010
LC209460	Hu/GII/JP/2010/GII.P16_GII.2/Kanagawa-50	Japan	2010
AB662875	Hu/GII.2/OC10009/2010/JP	Japan	2010

AB629941	Hu/Tokyo/10-14/2010/JPN	Japan	4/1/10
LC209467	Hu/GII/JP/2011/GII.P16_GII.2/Hokkaido-17	Japan	2011
LC209448	Hu/GII/JP/2011/GII.P16_GII.2/Osaka-9	Japan	2011
LC209479	Hu/GII/JP/2011/GII.P16_GII.2/Ehime-45	Japan	2011
LC209451	Hu/GII/JP/2011/GII.P16_GII.2/Osaka-18	Japan	2011
LC209447	Hu/GII/JP/2011/GII.P16_GII.2/Saitama-51	Japan	2011
LC209449	Hu/GII/JP/2011/GII.P16_GII.2/Osaka-26	Japan	2011
LC209468	Hu/GII/JP/2011/GII.P16_GII.2/Yamaguchi-4	Japan	2011
LC209471	Hu/GII/JP/2011/GII.P16_GII.2/Hiroshima-26	Japan	2011
LC209453	Hu/GII/JP/2011/GII.P16_GII.2/Osaka-023	Japan	2011
LC209452	Hu/GII/JP/2011/GII.P16_GII.2/Osaka-038	Japan	2011
KJ407074	Hu/GII.2/HS255/2011/USA	United States	1/30/11
AB629946	Hu/Tokyo/10-4320/2011/JPN	Japan	2/4/11
KC464505	Hu/GII.2/CGMH47/2011/TW	Taiwan	4/12/11
LC209466	Hu/GII/JP/2012/GII.P16_GII.2/Hokkaido-18	Japan	2012
LC145794	Hu/GII.2/Hiroshimacity2_2012_JP	Japan	2012
LC145786	Hu/GII.2/Akita8_2012_JP	Japan	2012
LC145788	Hu/GII.2/Niigata5_2012_JP	Japan	2012
LC209433	Hu/GII/JP/2012/GII.P16_GII.2/Tochigi-26	Japan	2012
LC145795	Hu/GII.2/Hiroshimacity1_2012_JP	Japan	2012
LC145796	Hu/GII.2/Ehime1_2012_JP	Japan	2012
LC145791	Hu/GII.2/Saitama4_2012_JP	Japan	2012
LC145789	Hu/GII.2/Fukui1_2012_JP	Japan	2012
LC209432	Hu/GII/JP/2012/GII.P16_GII.2/Tochigi-30	Japan	2012
LC145793	Hu/GII.2/Osakacity5_2012_JP	Japan	2012
LC145797	Hu/GII.2/Ehime2_2012_JP	Japan	2012
LC209445	Hu/GII/JP/2012/GII.P16_GII.2/Saitama-122	Japan	2012
LC145792	Hu/GII.2/Saitama5_2012_JP	Japan	2012
LC145790	Hu/GII.2/Fukui2_2012_JP	Japan	2012
LC209478	Hu/GII/JP/2012/GII.P16_GII.2/Ehime-46	Japan	2012
LC209446	Hu/GII/JP/2012/GII.P16_GII.2/Saitama-121	Japan	2012
LC145787	Hu/GII.2/Miyagi1_2012_JP	Japan	2012
LC209455	Hu/GII/JP/2013/GII.P16_GII.2/Miyagi-8	Japan	2013
LC209431	Hu/GII/JP/2013/GII.P16_GII.2/Tochigi-46	Japan	2013
LC209476	Hu/GII/JP/2013/GII.P16_GII.2/Ehime-8	Japan	2013
LC209475	Hu/GII/JP/2013/GII.P16_GII.2/Ehime-9	Japan	2013
LC209477	Hu/GII/JP/2013/GII.P16_GII.2/Ehime-6	Japan	2013
LC209456	Hu/GII/JP/2013/GII.P16_GII.2/Miyagi-7	Japan	2013
LC209444	Hu/GII/JP/2013/GII.P16_GII.2/Saitama-123	Japan	2013
LC209443	Hu/GII/JP/2013/GII.P16_GII.2/Saitama-124	Japan	2013
LC209442	Hu/GII/JP/2013/GII.P16_GII.2/Saitama-125	Japan	2013
LC145801	Hu/GII.2/Miyagi2_2014_JP	Japan	2014
LC145798	Hu/GII.2/Aomori7_2014_JP	Japan	2014
LC145800	Hu/GII.2/Akita8_2014_JP	Japan	2014
LC145799	Hu/GII.2/Akita7_2014_JP	Japan	2014
LC145807	Hu/GII.2/Ehime4_2014_JP	Japan	2014
LC145808	Hu/GII.2/Ehime5_2014_JP	Japan	2014
LC209458	Hu/GII/JP/2014/GII.P16_GII.2/Kanagawa-52	Japan	2014

LC145804	Hu/GII.2/Hiroshima6_2014_JP	Japan	2014
LC145805	Hu/GII.2/Hiroshimacity5_2014_JP	Japan	2014
LC209470	Hu/GII/JP/2014/GII.P16_GII.2/Hiroshima-30	Japan	2014
LC145806	Hu/GII.2/Hiroshimacity6_2014_JP	Japan	2014
LC209450	Hu/GII/JP/2014/GII.P16_GII.2/Osaka-225	Japan	2014
LC145803	Hu/GII.2/Osaka5_2014_JP	Japan	2014
LC228948	Hu/GII/JP/2014/GII.P16_GII.2/Osaka246	Japan	2014
LC145802	Hu/GII.2/Osaka4_2014_JP	Japan	2014
LC209441	Hu/GII/JP/2014/GII.P16_GII.2/Saitama-126	Japan	2014
LC209434	Hu/GII/JP/2014/GII.P16_GII.2/Tochigi-17	Japan	2014
LC209439	Hu/GII/JP/2014/GII.Pe_GII.2/Saitama-127	Japan	2014
KY457721	14-BI-1	Taiwan	2014/10
KY457722	14-BL-1	Taiwan	2014/11
LC209457	Hu/GII/JP/2015/GII.P2_GII.2/Miyagi-63	Japan	2015
LC209440	Hu/GII/JP/2015/GII.P2_GII.2/Saitama-169	Japan	2015
KY457724	15-BZ-1	Taiwan	2015/3
KY457725	15-CH-1	Taiwan	2015/3
KT962983	15-DS-4/2015/GII.2	Taiwan	2015/5
LC213885	Hu/GII/JP/2015/GII.P16-GII.2/Ibaraki197	Japan	6/17/15
KY421128	JS121062	China	2016
KY421127	JS1612108	China	2016
KY421126	JS120971	China	2016
KY421123	BJSCY	China	2016
KY421125	BJLJB	China	2016
KY421141	HLWL6	China	2016
LC215415	Hu/GII/JP/2016/GII.P16-GII.2/Kawasaki121	Japan	2016
KY421156	SC3	China	2016
LC215413	Hu/GII/JP/2016/GII.P16-GII.2/Kawasaki129	Japan	2016
LC215414	Hu/GII/JP/2016/GII.P16-GII.2/Kawasaki151	Japan	2016
LC279236	Hu/GII.P16-GII.2/330024/Tokyo/2016/JPN	Japan	2016
LC279234	Hu/GII.P16-GII.2/330022/Tokyo/2016/JPN	Japan	2016
LC279238	Hu/GII.P16-GII.2/330026/Tokyo/2016/JPN	Japan	2016
LC279235	Hu/GII.P16-GII.2/330023/Tokyo/2016/JPN	Japan	2016
KY421146	CQ22	China	2016
KY421154	BJ10	China	2016
KY421147	CQ23	China	2016
KY677827	GII/Hu/HK/2016/GII.2/CUHK-NS-934	Hong Kong	3/18/16
KY677828	GII/Hu/HK/2016/GII.P16-GII.2/CUHK-NS-1024	Hong Kong	8/25/16
KY905336	Hu/GII.P16_GII.2/Brisbane/QLDB411/2016/AU	Australia	2016/9
KY905337	Hu/GII.P16_GII.2/Brisbane/QLDB512/2016/AU	Australia	2016/9
LC325213	Hu/GII/JP/2016/GII.P16-GII.2/AichiF75	Japan	9/13/16
KY677829	GII/Hu/HK/2016/GII.P16-GII.2/CUHK-NS-1075	Hong Kong	9/17/16
KY677830	GII/Hu/HK/2016/GII.P16-GII.2/CUHK-NS-1082	Hong Kong	9/21/16
KY771081	CUHK-NS-1082	Hong Kong	9/21/16
MG746092	GZ20435	China	9/21/16
MG746047	SZ133	China	9/30/16
KY905338	Hu/GII.P16_GII.2/Brisbane/QLDB614/2016/AU	Australia	2016/10
KY457728	16-HS-1	Taiwan	2016/10

KY457729	16-HT-1	Taiwan	2016/10
KY817742	CUHK-NS-1120	Hong Kong	10/5/16
MG746046	SZ123	China	10/9/16
MG746060	SZ227	China	10/10/16
MG746061	SZ229	China	10/10/16
MG746059	SZ222	China	10/10/16
MG746035	SZ136	China	10/10/16
KY817743	CUHK-NS-1141	Hong Kong	10/11/16
LC213886	Hu/GII/JP/2016/GII.P16-GII.2/Ibaraki253	Japan	10/13/16
LC213887	Hu/GII/JP/2016/GII.P16-GII.2/Ibaraki267	Japan	10/29/16
MG746049	SZ153	China	11/1/16
KY407221	Hu/016R05/ZS/GD/CHN/2016	China	2016/11
KY407219	Hu/016R03/ZS/GD/CHN/2016	China	2016/11
KY407217	Hu/016R01/ZS/GD/CHN/2016	China	2016/11
KY407218	Hu/016R02/ZS/GD/CHN/2016	China	2016/11
KY407220	Hu/016R04/ZS/GD/CHN/2016	China	2016/11
KY485115	16SF2025_GII.2_Guangdong_CHN_2016	China	2016/11
KY485116	16SF2026_GII.2_Guangdong_CHN_2016	China	2016/11
KY457734	16-IP-1	Taiwan	2016/11
MG746101	GZ23200	China	11/4/16
LC213888	Hu/GII/JP/2016/GII.P16-GII.2/Ibaraki273	Japan	11/5/16
LC325214	Hu/GII/JP/2016/GII.P16-GII.2/AichiF120	Japan	11/7/16
MG746048	SZ150	China	11/11/16
MG746105	HNWL7	China	11/11/16
LC213889	Hu/GII/JP/2016/GII.P16-GII.2/Ibaraki290	Japan	11/11/16
KY677831	GII/Hu/HK/2016/GII.P16-GII.2/CUHK-NS-1219	Hong Kong	11/14/16
KY677832	GII/Hu/HK/2016/GII.P16-GII.2/CUHK-NS-1221	Hong Kong	11/15/16
MG746099	GZ24145	China	11/15/16
MG746100	GZ24139	China	11/15/16
MG746098	GZ24140	China	11/15/16
LC213891	Hu/GII/JP/2016/GII.P16-GII.2/Ibaraki329	Japan	11/15/16
KY677833	GII/Hu/HK/2016/GII.P16-GII.2/CUHK-NS-1222	Hong Kong	11/15/16
LC213890	Hu/GII/JP/2016/GII.P16-GII.2/Ibaraki324	Japan	11/16/16
MG746087	CQ24	China	11/18/16
MG746088	CQ25	China	11/18/16
KY421044	Hu/GII.2/CUHK-NS-1231/HKG/2016	Hong Kong	11/19/16
LC213892	Hu/GII/JP/2016/GII.P16-GII.2/Ibaraki374	Japan	11/20/16
MG746014	GZ24890	China	11/21/16
MG746096	GZ24892	China	11/21/16
MG746097	GZ24891	China	11/21/16
LC325215	Hu/GII/JP/2016/GII.P16-GII.2/AichiF149	Japan	11/22/16
MG746050	SZ170	China	11/23/16
MG746095	GZ25269	China	11/25/16
LC213894	Hu/GII/JP/2016/GII.P16-GII.2/Ibaraki423	Japan	11/25/16
MG746036	SZ190	China	11/25/16
LC213893	Hu/GII/JP/2016/GII.P16-GII.2/Ibaraki412	Japan	11/25/16
LC325216	Hu/GII/JP/2016/GII.P16-GII.2/AichiF158	Japan	11/28/16
MG746090	CQ28	China	11/28/16

MG746091	CQ30	China	11/28/16
MG746089	CQ27	China	11/28/16
MG746052	SZ177	China	11/30/16
MG746051	SZ176	China	11/30/16
KY865306	Hu/USA/2016/GII.P16-GII.2/Santa Rosa 1764	United States	11/30/16
MG746008	CQ031	China	11/30/16
MG746136	CQ032	China	11/30/16
MG746083	CQ3	China	11/30/16
MG746135	CQ030	China	11/30/16
MG746056	SZ200	China	11/30/16
MG746037	SZ205	China	11/30/16
MG746063	SZ248	China	12/1/16
KY485118	16SF2153_GII.2_Guangdong_CHN_2016	China	2016/12
KY485117	16SF2151_GII.2_Guangdong_CHN_2016	China	2016/12
KY485124	16F2152_GII.2_Guangdong_CHN_2016	China	2016/12
KY457586	Hu/016T03/ZS/GD/CHN/2016	China	2016/12
KY457584	Hu/016T01/ZS/GD/CHN/2016	China	2016/12
KY457585	Hu/016T02/ZS/GD/CHN/2016	China	2016/12
KY485119	16SF2285_GII.2_Guangdong_CHN_2016	China	2016/12
KY485121	16SF2030_GII.2_Guangdong_CHN_2016	China	2016/12
KY485120	16SF2029_GII.2_Guangdong_CHN_2016	China	2016/12
KY485125	16F2149_GII.2_Guangdong_CHN_2016	China	2016/12
KY485126	16F2161_GII.2_Guangdong_CHN_2016	China	2016/12
KY457580	Hu/016S01/ZS/GD/CHN/2016	China	2016/12
KY457581	Hu/016S02/ZS/GD/CHN/2016	China	2016/12
KY457583	Hu/016S04/ZS/GD/CHN/2016	China	2016/12
KY457582	Hu/016S03/ZS/GD/CHN/2016	China	2016/12
MG746027	CQ1	China	12/1/16
MG746062	SZ243	China	12/1/16
MG763365	GII/Hu/2016/CHN/GII.P16-GII.2/HuzhouNS16482	China	2016/12
KY485123	16SF21134_GII.2_Guangdong_CHN_2016	China	2016/12
KY485122	16SF21132_GII.2_Guangdong_CHN_2016	China	2016/12
MG746054	SZ185	China	12/2/16
MG746053	SZ184	China	12/2/16
MG746077	BJFTsmq	China	12/2/16
MG746004	FJ16235609	China	12/2/16
MG746003	FJ16235606	China	12/2/16
MG746106	FJ16235607	China	12/2/16
LC213895	Hu/GII/JP/2016/GII.P16-GII.2/Ibaraki472	Japan	12/2/16
MG746057	SZ208	China	12/2/16
MG746058	SZ211	China	12/2/16
LC213896	Hu/GII/JP/2016/GII.P16-GII.2/Ibaraki518	Japan	12/3/16
MG746108	BJHDG3	China	12/4/16
MG746084	CQ5	China	12/5/16
LC213897	Hu/GII/JP/2016/GII.P16-GII.2/Ibaraki536	Japan	12/5/16
MG746107	BJHD1608Y123	China	12/5/16
MG746104	HNWL6	China	12/6/16
LC213898	Hu/GII/JP/2016/GII.P16-GII.2/Ibaraki602	Japan	12/7/16

MG746085	CQ6	China	12/7/16
MG746002	JSNJ121057	China	12/9/16
MG746079	JSNJ120972	China	12/9/16
KY421121	JS1208	China	12/10/16
MG746001	JSNJ12108	China	12/10/16
MG746081	JSNJ121062	China	12/10/16
KY817744	CUHK-NS-1245	Hong Kong	12/10/16
LC213900	Hu/GII/JP/2016/GII.P16-GII.2/Ibaraki636	Japan	12/11/16
LC213899	Hu/GII/JP/2016/GII.P16-GII.2/Ibaraki607	Japan	12/11/16
MG746066	SZ267	China	12/11/16
MG746065	SZ264	China	12/11/16
KY421122	BJSMQ	China	12/12/16
MG746103	HNCD7	China	12/12/16
MG746093	GZ27191	China	12/12/16
MG746094	GZ27189	China	12/12/16
MG746015	GZ27188	China	12/12/16
MG746086	CQ13	China	12/13/16
LC213901	Hu/GII/JP/2016/GII.P16-GII.2/Ibaraki658	Japan	12/14/16
MG746147	HNCD2503	China	12/15/16
MG746064	SZ260	China	12/16/16
MG746102	SC5	China	12/16/16
MG746029	SC1	China	12/16/16
MG746072	SZ311	China	12/19/16
MG746134	CQ022	China	12/20/16
MG746078	BJFT10	China	12/20/16
MG746073	SZ314	China	12/20/16
MG746074	SZ319	China	12/20/16
KY865307	Hu/USA/2016/GII.P16-GII.2/Nashville 2122	United States	12/21/16
MG746137	CQ034	China	12/21/16
MG746009	CQ035	China	12/21/16
MG746067	SZ285	China	12/21/16
MG746069	SZ292	China	12/22/16
MG746068	SZ289	China	12/22/16
MG746140	CQ043	China	12/22/16
MG746139	CQ042	China	12/22/16
MG746141	CQ044	China	12/22/16
MG746124	GZ28589	China	12/23/16
MG746123	GZ28577	China	12/23/16
MG745998	GZ28587	China	12/23/16
MG746071	SZ307	China	12/23/16
MG746133	CQ010	China	12/23/16
KY817745	CUHK-NS-1252	Hong Kong	12/23/16
MG746070	SZ299	China	12/24/16
KY817746	CUHK-NS-1261	Hong Kong	12/25/16
KY817747	CUHK-NS-1263	Hong Kong	12/25/16
MG745999	GZ28626	China	12/26/16
MG746125	GZ28621	China	12/26/16
MG746122	GZ28491	China	12/27/16

MG746121	GZ28487	China	12/27/16
MG746016	HNCD2701	China	12/29/16
MG746149	HNCD2704	China	12/29/16
MG746150	HNCD2708	China	12/29/16
MG746167	SZ05	China	12/29/16
MG746076	SZ325	China	12/29/16
MG746075	SZ322	China	12/29/16
MG746000	GZ29003	China	12/30/16
MG746128	GZ29009	China	12/30/16
MG746129	Z29010	China	12/30/16
MG746130	CQ001	China	1/1/17
MG746112	GZ1520	China	1/4/17
MG745988	GZ1519	China	1/4/17
MG746142	GX170034-1	China	1/4/17
MG746143	GX170034-4	China	1/4/17
MG746115	GZ1617	China	1/5/17
MG746111	GZ1500	China	1/5/17
MG746114	GZ1614	China	1/5/17
KY806296	Hu/JSYZ201701-1/CHN	China	1/5/17
MG746132	CQ009	China	1/5/17
MG746131	CQ007	China	1/5/17
KY806300	Hu/JSNJ201701-1/CHN	China	1/5/17
MG745991	GZ1619	China	1/5/17
MG746117	GZ1621	China	1/5/17
MG745990	GZ1528	China	1/5/17
MG746116	GZ1620	China	1/5/17
MG746007	CQ006	China	1/5/17
MG746169	SZ20	China	1/7/17
MG746168	SZ08	China	1/7/17
MG745993	GZ2043	China	1/10/17
MG745994	GZ2113	China	1/10/17
MG745985	GZ60	China	1/10/17
MG746110	GZ78	China	1/10/17
MG746109	GZ72	China	1/10/17
MG746120	GZ2125	China	1/10/17
MG746145	JSYZ32	China	1/11/17
MG746144	JSYZ25	China	1/11/17
MG746146	JSYZ35	China	1/11/17
KY817748	CUHK-NS-1299	Hong Kong	1/12/17
KY817749	CUHK-NS-1305	Hong Kong	1/16/17
KY817750	CUHK-NS-1312	Hong Kong	1/18/17
MG763366	GII/Hu/2017/CHN/GII.P16-GII.2/HuzhouNS17057	China	2017/2
MG763367	GII/Hu/2017/CHN/GII.P16-GII.2/HuzhouNS17072	China	2017/2
MG763354	GII/Hu/2017/CHN/GII.P16-GII.2/HuzhouN17174	China	2017/2
KY817751	CUHK-NS-1337	Hong Kong	2/6/17
KY817752	CUHK-NS-1344	Hong Kong	2/10/17
MG746032	SZ45	China	2/13/17
KY817753	CUHK-NS-1349	Hong Kong	2/13/17

MG746171	SZ36	China	2/15/17
MG746170	SZ30	China	2/15/17
KY806291	Hu/JSCZ201702-2/CHN	China	2/15/17
KY806290	Hu/JSCZ201702-1/CHN	China	2/15/17
MG746172	SZ40	China	2/16/17
KY817754	CUHK-NS-1354	Hong Kong	2/17/17
KY806292	Hu/JSWX201702-1/CHN	China	2/17/17
KY806293	Hu/JSWX201702-2/CHN	China	2/17/17
MG746176	SZ90	China	2/19/17
MG746177	SZ95	China	2/20/17
KY806298	Hu/JSSZ201702-1/CHN	China	2/21/17
MG746178	SZ106	China	2/21/17
MG746151	SHDJN30	China	2/21/17
MG746174	SZ72	China	2/23/17
KY806297	Hu/JSYC201702-1/CHN	China	2/23/17
MG746158	BJSYL48	China	2/23/17
MG746157	BJSYL45	China	2/23/17
KY806301	Hu/JSTZ201702-1/CHN	China	2/24/17
KY806299	Hu/JSNT201702-1/CHN	China	2/24/17
MG746153	JSWXCYY	China	2/24/17
MG746156	JSWXJINYQ	China	2/24/17
MG746155	JSWXCHYY	China	2/24/17
MG746154	JSWXCHLJ	China	2/24/17
MG746159	BJSYL53	China	2/24/17
MG746180	SZ122	China	2/26/17
MG746034	SZ127	China	2/26/17
MG746179	SZ121	China	2/26/17
MG746152	SHDJN39	China	2/27/17
MH068804	Enshi/Hubei019/China/2017	China	2017/3
MH068807	Enshi/Hubei024/China/2017	China	2017/3
MH068805	Enshi/Hubei021/China/2017	China	2017/3
MH068808	Enshi/Hubei025/China/2017	China	2017/3
MH068803	Enshi/Hubei018/China/2017	China	2017/3
MH068801	Enshi/Hubei015/China/2017	China	2017/3
MH068802	Enshi/Hubei017/China/2017	China	2017/3
MH068806	Enshi/Hubei023/China/2017	China	2017/3
MH068810	Enshi/Hubei016/China/2017	China	2017/3
MH068793	QJ/Hubei04/China/2017	China	2017/3
MH068798	QJ/Hubei010/China/2017	China	2017/3
MH068792	QJ/Hubei03/China/2017	China	2017/3
MH068795	QJ/Hubei06/China/2017	China	2017/3
MH068800	QJ/Hubei013/China/2017	China	2017/3
MH068796	QJ/Hubei08/China/2017	China	2017/3
MH068794	QJ/Hubei05/China/2017	China	2017/3
MH068791	QJ/Hubei01/China/2017	China	2017/3
MH068799	QJ/Hubei012/China/2017	China	2017/3
MH068797	QJ/Hubei09/China/2017	China	2017/3
MG763355	GII/Hu/2017/CHN/GII.P16-GII.2/HuzhouN17625	China	2017/3

MG763356	GII/Hu/2017/CHN/GII.P16-GII.2/HuzhouN17626	China	2017/3
MG763372	GII/Hu/2017/CHN/GII.P16-GII.2/HuzhouNS17135	China	2017/3
MH068815	HG/Hubei042/China/2017	China	2017/3
MH068820	HG/Hubei059/China/2017	China	2017/3
MH068812	HG/Hubei030/China/2017	China	2017/3
MH068816	HG/Hubei048/China/2017	China	2017/3
MH068817	HG/Hubei049/China/2017	China	2017/3
MH068813	HG/Hubei032/China/2017	China	2017/3
MH068814	HG/Hubei036/China/2017	China	2017/3
MH068811	HG/Hubei027/China/2017	China	2017/3
MH068819	HG/Hubei054/China/2017	China	2017/3
MH068818	HG/Hubei050/China/2017	China	2017/3
MG763368	GII/Hu/2017/CHN/GII.P16-GII.2/HuzhouNS17116	China	2017/3
MG763371	GII/Hu/2017/CHN/GII.P16-GII.2/HuzhouNS17123	China	2017/3
MG763370	GII/Hu/2017/CHN/GII.P16-GII.2/HuzhouNS17122	China	2017/3
MG763369	GII/Hu/2017/CHN/GII.P16-GII.2/HuzhouNS17118	China	2017/3
MH068809	Enshi/Hubei020/China/2017	China	2017/3
MG763357	GII/Hu/2017/CHN/GII.P16-GII.2/HuzhouN17636	China	2017/3
MG763358	GII/Hu/2017/CHN/GII.P16-GII.2/HuzhouN17638	China	2017/3
MG746163	LNSY3	China	3/5/17
MG746166	LNSY10	China	3/5/17
MG746165	LNSY7	China	3/5/17
MG746164	LNSY5	China	3/5/17
MG746175	SZ83	China	3/6/17
MG746006	BJSYL75	China	3/7/17
MG746161	BJSYL72	China	3/8/17
MG746160	BJSYL71	China	3/8/17
KY806294	Hu/JSWX201703-1/CHN	China	3/10/17
MG746185	LNSY102	China	3/17/17
MF167650	Hu/JSCZ201703-19/CHN	China	3/18/17
MF167652	Hu/JSWX201703-10/CHN	China	3/20/17
MF167651	Hu/JSCZ201703-22/CHN	China	3/20/17
MG746183	SZ196	China	3/30/17
MG746182	SZ191	China	3/30/17
MG763359	GII/Hu/2017/CHN/GII.P16-GII.2/HuzhouN17645	China	2017/4
MG763360	GII/Hu/2017/CHN/GII.P16-GII.2/HuzhouN17647	China	2017/4
MG763361	GII/Hu/2017/CHN/GII.P16-GII.2/HuzhouN17648	China	2017/4
MG746024	HNLD01	China	4/6/17
MG746039	HNXT01	China	4/10/17
MG746193	HNXT11	China	4/10/17
MG746201	BJFT345	China	4/14/17
MG746041	BJFT359	China	4/14/17
MG746191	LNDL00402	China	4/15/17
MG746025	LNDL00401	China	4/15/17
MG746212	BJFT371	China	4/16/17
MG746213	BJFT385	China	4/17/17
MG746214	BJFT408	China	4/18/17
MG746215	BJFT423	China	4/20/17

MG746184	LNSY112	China	4/23/17
MG746186	LNSY120	China	4/24/17
MG746217	BJFT465	China	4/28/17
MG746216	BJFT463	China	4/28/17
MG763373	GII/Hu/2017/CHN/GII.P16-GII.2/HuzhouNS17158	China	2017/5
MG763363	GII/Hu/2017/CHN/GII.P16-GII.2/HuzhouN17663	China	2017/5
MG763362	GII/Hu/2017/CHN/GII.P16-GII.2/HuzhouN17653	China	2017/5
MG746220	BJFT497	China	5/5/17
MG746218	BJFT477	China	5/5/17
MG746219	BJFT485	China	5/5/17
MG746221	BJFT521	China	5/6/17
MG746222	BJFT527	China	5/6/17
MG746223	BJFT530	China	5/6/17
MG746224	BJFT536	China	5/7/17
MG746225	BJFT540	China	5/7/17
MG746211	BJFT545	China	5/13/17
MG746210	BJFT566	China	5/13/17
MG746209	BJFT574	China	5/15/17
MG746208	BJFT593	China	5/16/17
MG746196	JNSY2	China	5/17/17
MG746195	JNSY5	China	5/18/17
MG746187	LNJZ201	China	5/18/17
MG746194	JNSY10	China	5/18/17
MG746188	LNJZ301	China	5/19/17
MG746198	JNCH4	China	5/20/17
MG746199	JNCH2	China	5/20/17
MG746197	JNCH9	China	5/20/17
MG746019	JNCH8	China	5/20/17
MG746020	JN-II8	China	5/20/17
MG746044	BJFT640	China	5/26/17
MG746205	BJFT668	China	5/31/17
MG746206	BJFT664	China	5/31/17
MG746189	LNJZ311	China	5/31/17
MG746204	BJFT698	China	6/8/17
MG746203	BJFT694	China	6/8/17
MG746200	BJFT704	China	6/12/17
MG746026	LNLY502	China	6/13/17
MG763374	GII/Hu/2017/CHN/GII.P16-GII.2/HuzhouNS17442	China	2017/11
MG763375	GII/Hu/2017/CHN/GII.P16-GII.2/HuzhouNS17449	China	2017/11
MG763364	GII/Hu/2017/CHN/GII.P16-GII.2/HuzhouN17790	China	2017/11
MG763376	GII/Hu/2017/CHN/GII.P16-GII.2/HuzhouNS17460	China	2017/11
MG763377	GII/Hu/2017/CHN/GII.P16-GII.2/HuzhouNS17466	China	2017/11
