# Supplementary material for: Molecular Evolution of Human Norovirus GII.2 Clusters
Source: Front Microbiol. 2021 Mar 22;12:655567. doi: 10.3389/fmicb.2021.655567 (PMC8019798; doi:10.3389/fmicb.2021.655567)
Supplement: Supplementary file 3 [file Table_2.pdf]

Supplementary Table S2. Classification and sampling year of human norovirus GII.2 sequences used in the present study.

Cluster	n	Sampling year				
		1970-1979	1980-1989	1990-1999	2000-2009	2010-2017
I	4			1	3	
II	6				6	
III	21				21	
IV	25				12	13
V	64				6	58
VI	19					19
VII	4					4
VIII	357					357
Ungrouped	19	2	1	2	13	1
**Total**	**519**	**2**	**1**	**3**	**61**	**452**
