# Supplementary material for: Molecular Evolution of Human Norovirus GII.2 Clusters
Source: Front Microbiol. 2021 Mar 22;12:655567. doi: 10.3389/fmicb.2021.655567 (PMC8019798; doi:10.3389/fmicb.2021.655567)
Supplement: Supplementary file 4 [file Table_3.pdf]

Supplementary Table S3. Estimated time to the most recent common ancestor of each human norovirus GII.2 cluster.

HuNoV GII.2 cluster	Time to the most recent common ancestor in years (mean and 95% credibility interval)
I	1992.42 (1990.71, 1994.10)
II	1996.98 (1995.61, 1998.23)
III	2003.02 (2002.59, 2003.43)
IV	2005.29 (2004.40, 2006.06)
V	2000.18 (1998.84, 2001.42)
VI	2007.96 (2007.10, 2008.75)
VII	2009.13 (2007.47, 2010.59)
VIII	2012.39 (2011.42, 2013.44)
Full data set	1968.89 (1965.49, 1972.06)
