# Supplementary material for: Molecular Evolution of Human Norovirus GII.2 Clusters
Source: Front Microbiol. 2021 Mar 22;12:655567. doi: 10.3389/fmicb.2021.655567 (PMC8019798; doi:10.3389/fmicb.2021.655567)
Supplement: Supplementary file 5 [file Table_4.pdf]

Supplementary Table S4. BUSTED and RELAX analysis of selection for persistent lineages and highly supported phylogenetic clades I-VIII for *VP1* sequences of human norovirus GII.2 genotype.

BUSTED		RELAX									
Model	log(lik)	Model	log(lik)	I	II	III	IV	V	VI	VII	VIII
dN/dS > 1	-16779.1	Separate *k*	-16479.25	0.72	2.29	2.10	0.60	2.43	0.35	2.04	0.43
dN/dS = 1	-16780.8	*k* = 1	-16769.93								
LRT *p* -value	0.18	LRT *p* -value	0.04								
